# The health outcomes and costs of people attending an interdisciplinary chronic disease service in regional Australia: protocol for a longitudinal cohort investigation

**DOI:** 10.1186/1472-6963-13-410

**Published:** 2013-10-11

**Authors:** Zephanie Tyack, Kerrie-Anne Frakes, Petrea Cornwell, Suzanne S Kuys, Adrian G Barnett, Steven M McPhail

**Affiliations:** 1 Clinical Support Services, Central Queensland Hospital and Health Service, Rockhampton Hospital, Canning St, Rockhampton, QLD 4700, Australia; 2 School of Health and Rehabilitation Sciences, The University of Queensland, St Lucia, QLD 4067, Australia; 3 Allied Health Research Collaborative, Metro North Hospital and Health Service, Rode Rd, Chermside, Qld 4032, Australia; 4 Griffith Health Institute Behavioural Basis of Health Program, Griffith University, Messines Ridge Rd, Mt Gravatt, QLD 4172, Australia; 5 Centre for Musculoskeletal Research, Griffith Health Institute, Griffith University, Gold Coast Campus, Parklands Drive, Southport, QLD 4222, Australia; 6 Institute of Health and Biomedical Innovation and School of Public Health & Social Work, Queensland University of Technology, Victoria Park Road, Kelvin Grove, QLD 4059, Australia; 7 Centre for Functioning and Health Research, Metro South Hospital and Health Service, Corner of Ipswich Road and Cornwall Street, Buranda, QLD 4102, Australia

**Keywords:** Chronic disease, Multimorbidity, Interprofessional care, Rural health, Allied health services, Health costs, Health outcomes

## Abstract

**Background:**

Rates of chronic disease are escalating around the world. To date health service evaluations have focused on interventions for single chronic diseases. However, evaluations of the effectiveness of new intervention strategies that target single chronic diseases as well as multimorbidity are required, particularly in areas outside major metropolitan centres where access to services, such as specialist care, is difficult and where the retention and recruitment of health professionals affects service provision.

**Methods:**

This study is a longitudinal investigation with a baseline and three follow-up assessments comparing the health and health costs of people with chronic disease before and after intervention at a chronic disease clinic, in regional Australia. The clinic is led by students under the supervision of health professionals. The study will provide preliminary evidence regarding the effectiveness of the intervention, and evaluate the influence of a range of factors on the health outcomes and costs of the patients attending the clinic. Patients will be evaluated at baseline (intake to the service), and at 3-, 6-, and 12-months after intake to the service. Health will be measured using the SF-36 and health costs will be measured using government and medical record sources. The intervention involves students and health professionals from multiple professions working together to treat patients with programs that include education and exercise therapy programs for back pain, and Healthy Lifestyle programs; as well as individual consultations involving single professions.

**Discussion:**

Understanding the effect of a range of factors on the health state and health costs of people attending an interdisciplinary clinic will inform health service provision for this clinical group and will determine which factors need to be controlled for in future observational studies. Preliminary evidence regarding changes in health and health costs associated with the intervention will be a platform for future clinical trials of intervention effectiveness. The results will be of interest to teams investigating new chronic disease programs particularly for people with multimorbidity, and in areas outside major metropolitan centres.

**Trial registration:**

Australia and New Zealand Clinical Trials Registry: ACTRN12611000724976

## Background

A unique student-led interdisciplinary chronic disease health service was established in regional Australia in 2010 to address workforce shortages and spiralling rates of chronic disease in rural and regional Queensland. The service also aimed to improve local clinical placement options for allied health students. The service was established in a shopping centre with easy access using public transport.

Demographics of the people attending the service in the first year of the service (prior to commencement of the study) revealed very high rates of multimorbidity (97%) and higher rates of indigenous clients (7.1%) compared with the local community (5.9%) [1]. These demographics are relevant to the expected health outcomes of people attending the service as people with multimorbidity have worse health outcomes [2], and place a larger burden on the healthcare system than those with a single chronic disease. Additionally, indigenous Australians generally have worse health outcomes than non-indigenous Australians; including mortality and disability rates [3].

Interdisciplinary care has been promoted as a solution to maintain or improve the quality of healthcare, particularly in regional and rural areas [4-6] where health service disparity, healthcare access, and a worse burden of chronic diseases may place people living in these areas at a disadvantage in comparison to metropolitan areas [7]. Despite this, there are few high quality studies that evaluate the effectiveness of interdisciplinary care for chronic disease or primary care [8], even in metropolitan areas.

This paper describes the study protocol of a longitudinal evaluation of a unique interdisciplinary health service established in a regional Australian city to address chronic disease. The study will estimate the health outcomes and associated health costs of people attending the service.

## Methods

### Setting and services

**Figure 1:**
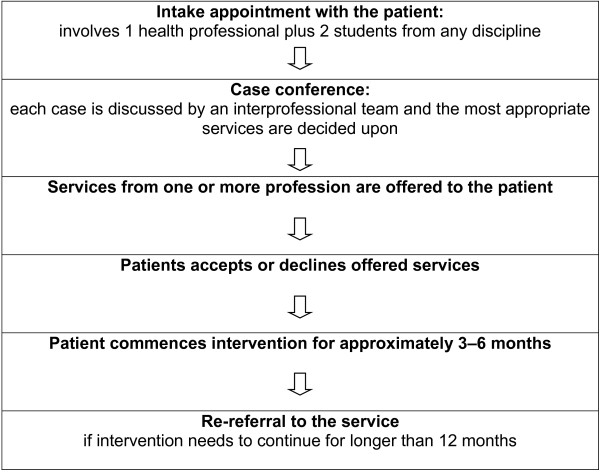
The process for receiving services at the interdisciplinary clinic.

The regional interdisciplinary chronic disease health service is located in a regional city (Rockhampton) approximately 700 kilometres from the state’s capital. Services are provided to people with chronic diseases in the regional city and surrounding rural and remote areas (providing services to more than 100,000 residents). Referral to the interdisciplinary clinic can arise from three sources: self referral, referral from the local hospital or referral from a general medical practitioner; although the majority (90%) are referred by their general practitioner [1]. Services are delivered by an interdisciplinary team of nurses and allied health professionals, including podiatrists, occupational therapists, exercise physiologists, a social worker, a speech pathologist, a dietician, a physiotherapist, a pharmacist and an indigenous health worker. Qualified professional supervisors in allied health disciplines supervise up to four students each who provide a range of services to clients with a wide range of chronic diseases that include hypertension, osteoarthritis, high cholesterol, diabetes, and chronic back pain. The process for obtaining services is shown in Figure 1.

### Study design

A longitudinal cohort study focusing on the health outcomes and costs of patients with chronic diseases attending the service will be implemented. Patients will be evaluated at baseline (intake to the service), and at 3-, 6-, and 12-months after intake to the service. The 3- and 6-month follow-ups were included to evaluate short-term outcomes after commencement of the intervention. The 12-month follow-up was included to evaluate intermediate outcomes to pick up a potential delay in the positive impact of intervention on some dimensions of health. This study has been prospectively registered with the Australian and New Zealand Clinical Trials Registry (ACTRN12611000724976).

### Study objectives

#### ***Aims***

1. To examine the effectiveness of the service up to 12-months post-intake in improving a range of health outcomes (including length of stay in hospital, number of admissions, health state, preference based health state, and health-related costs).

2. To examine the effect of a range of factors including comorbidities, sociodemographic factors, and type of intervention received, on the health state, preference-based health state, length of stay in hospital, number of admissions, and health-related costs of people attending the service up to 12-months post intake.

3. To determine the difference in health between clients attending the service before and after receiving intervention and the general population using Australian population normative data.

#### ***Hypotheses***

1. Health will be maintained or improve over time from intake (baseline) to 12-months after intake with a small average effect size anticipated for improvement (effect size ≤ 0.2).

2. Health outcomes will be moderately to strongly and negatively associated with the number of chronic diseases (effect size ≥ 0.5).

3. Days in hospital and inpatient admission costs will decrease after intake to the service compared with before intake to the service.

4. The health of clients attending the service will be worse than the general population using Australian population normative data at all times (effect size ≥ 0.2).

### Participants and selection criteria

All clients who attended the service will be eligible to participate. The following are inclusion criteria: presence of at least one chronic disease; aged 18 or older; and attendance at their first intake appointment. In addition, clients will be required to attend a minimum of one intervention session at the service for follow-up data to be included in analyses of clinic effectiveness over time. The following are exclusion criteria: a severe communication disability or behavioural disorder that would compromise the ability to participate in the intervention program or complete the questionnaires; a cognitive deficit that impairs the ability to read and write; and known pregnancy or up to 6-months postpartum that would influence body mass index (BMI), waist measurements, and possibly other outcomes. All participants receive individually tailored care prescribed by the service.

### Study measures

All study measures will be completed at the site of the interdisciplinary service at baseline. However, people who are unable to attend the clinic for a follow-up assessment will be posted self-report questionnaires. Questionnaires will be self-administered except in cases where it is clear that the patient is unable to complete the questionnaire due to an impairment (such as visual disturbance) that makes self-completion difficult. In these cases questionnaires will be interviewer-administered. Table 1 provides a summary of each of the measures at each time point.

**Table 1:** Study outcome measures at each follow-up time

**Baseline**	**3 months**	**6 months**	**12 months**
SF-36	SF-36	SF-36	SF-36
BMI and waist measurement	BMI and waist measurement	BMI and waist measurement	BMI and waist measurement
Comorbiditymeasure	Comorbidity measure	Comorbidity measure	Comorbidity measure
K6			
Medical Outcomes Study Social Support Survey			
TUG	TUG	TUG	TUG
Self-reported health perception question	Self-reported health perception question	Self-reported health perception question	Self-reported health perception question
Sociodemographic information			
	Intervention data	Intervention data	Intervention data

### Health outcomes

Health will be measured using the Australian SF-36 (Version 1). The SF-36 is a 36-item generic measure of health with eight scales including physical functioning, role limitations, bodily pain, social functioning, general mental health, role limitations, vitality, and general health perceptions [9]. Two summary scores are obtained from these scales: a physical components summary and mental components summary. Extensive psychometric testing has occurred using people with a wide range of chronic diseases or single chronic diseases [10-13]. A review of studies has demonstrated that the SF-36 is superior in terms of sensitivity to change and responsiveness compared to other measures of health [9]. In addition, the SF-36 has been shown to discriminate between types and severities of chronic diseases and to distinguish between people with a chronic medical condition only, from those with a chronic medical condition plus a psychiatric disorder [14]. A preference-based measure of health will be derived from the SF-36 to produce a summary health score (multi-attribute utility score) for individuals at each time point.

Hospital utilisation will be measured using the number of days spent in hospital and number of hospital admissions during the one-year follow-up as indicators for intervention effectiveness. This will be accessed from hospital records for each participant.

### Healthcare costs

Healthcare costs will be determined using Medicare and hospital utilisation records. Medicare data (including Pharmaceutical Benefits Scheme data) will be accessed for the 12-months prior and 12-months following intake (baseline), which will permit estimation of within-patient change in healthcare costs. This data will allow costing of Hospital, Medicare and Pharmaceutical Benefits Scheme related items that will include inpatient admission costs, out-of-pocket expenses for general practitioners and specialist medical appointments, and out-of-pocket medication related expenses. Clinic health professional labour costs related to delivering the interdisciplinary intervention to study participants will also be recorded. Expenses related to alternative medications will not be captured by these data sources.

### Anthropometric measures

Height and weight measurements will be taken to allow BMI to be calculated. A waist measurement will also be taken as an indicator of obesity [15]. Height will be measured without shoes to the nearest 0.1 cm using a stadiometer (Wedderburn Portable Height Rod, Model WS-HRP). The participant will be required to stand with their heels touching the vertical foot panel of the stadiometer and the participant’s head held in the Frankfort plane. Weight will be measured in bare feet or socks (without shoes) using a calibrated balance scale (Tanita Digital Scales, Model HD-351) with the participant dressed in light clothing, and any clothing pockets emptied. Waist circumference will be measured to the nearest 0.1 cm equidistance between the last palpable rib and the top of the superior border of the iliac crest with the tape perpendicular to long axis of body and parallel to ground at the end of expiration [16,17]. The patient will be requested to place their arms relaxed by their side for this measurement. A narrow 7 mm wide flexible steel anthropometrical tape measure (Rosscraft) will be used.

### Psychological distress or well-being

Psychological distress or well-being (including mood and anxiety disorders) will be screened for using the 6-item Kessler6 (K6). A unidimensional factor structure has been supported in a large Australian community population [18] and the validity of the K6 has been supported for screening serious mental illness [19] as well as for discriminating between people with and without psychological distress in the community [20].

### Disease burden and number and type of comorbid conditions

Disease burden and the number and type of comorbid conditions will be measured using a self-report comorbidity measure [21]. Using the comorbidity measure [21] 25 common chronic diseases (excluding depression) are rated for the degree to which each condition interferes with daily activities. Scores on this measure have been found to correlate more strongly with health state measured using the SF-36 than other comorbidity indices in community samples [22,23].

### Perceived functional and structural social support

Perceived functional and structural social support will be measured using the Medical Outcomes Study Social Support Survey [24]. This measure was designed for research with people with chronic conditions [9] and testing by the authors has supported validity for use among people with chronic diseases including support for four social support subscales [24].

### Physical function

Physical function will be measured using the Timed Up and Go Test (TUG) as an objective measure of functional mobility [25]. Psychometric testing has supported use of the TUG as a measure of functional mobility in older adults [26-28]. Responsiveness of the TUG has been supported in a study of geriatric patients attending day hospital [29] and sensitivity to change has been supported among people with chronic diseases [30,31].

### Self-reported health perception

Self-reported health perception will be measured using a single question where participants rate their health on a 1 to 5 point scale as excellent to poor respectively [32]. Strong support exists for the ability of a single question regarding self-reported health perception to reflect the current, changing and future health status of people with chronic disease [32].

### Sociodemographic and intervention data

Demographic data collected only at baseline will include: age, gender, indigenous status, country of birth, language (other than English), marital status, home ownership, highest level of education, occupation, and employment status. Home ownership, highest level of education and occupation will be used as socioeconomic indicators. Intervention data collected will include the length and type of intervention received (for example, back school program, gym program, diabetes education, or the professional seen).

### Sample size and power

A minimum sample size of 130 participants is required to examine the change over time from baseline to 12-months post-intake of the SF-36; using a one-group longitudinal design, a power of 80% with a two-sided type 1 error of 5% to detect a difference of at least 5 points in the social functioning scale of the SF-36 using a t-test [33]. Three to five points of change in SF-36 scores has been reported as the minimally clinically important difference [33,34] and is equivalent to a small effect size for those with chronic diseases [34]. A small effect size is anticipated for changes in health over 12-months based on the small to large effect sizes found in treatment effectiveness studies using the SF-36 in related chronic disease studies over similar times [7,12,34,35] and the high percentage of clients with multiple chronic diseases attending the service prior to the study commencement, for whom moderate to large effect sizes are unlikely. The sample size calculation was based on the social functioning scale of the SF-36 as this outcome would likely show the smallest difference and therefore need the largest sample size. Therefore, provided the study sample size exceeds 130 participants, this investigation should have greater than 80% power for all outcomes.

### Data analysis

Descriptive statistics and exploratory plots (boxplots or histograms) will be used to describe the outcome variables (e.g., health state, preference based measure of health, hospital utilisation, and costs) and potential predictors (e.g., age, employment status).

SF-36 scale raw scores will be transformed to a 0 to 100 scale. Results will be analysed using the 8 scales and the summary scores where appropriate. Scatterplots of the relationship between variables will be examined to check for linearity or potentially non-linear associations. Mean SF-36 scores will be plotted over time by demographic and disease subgroups to visually examine chronic disease progression. Regression models will be used to examine the effect of time and predictors on post-intake SF-36 scores. The scores will be modelled at 3- to 12-months with the baseline score as a predictor in order to: i) analyse the change from baseline; ii) help control for regression to the mean [36]. Mixed models will be used with subject-specific intercepts to model the dependence in results over time from the same subject. The lasso technique will be used to choose the best set of predictor variables from an initially large set of potentially important variables [37]. The residual distribution of the best model will be examined to check that it is approximately normally distributed with no large outliers. The count outcome variables (i.e., cost, days in hospital, and number of hospitalisations) will be analysed using the model described above but assuming a Poisson distribution.

The predictors to be examined in all of the models will be: BMI and waist measurement, disease burden, number and type of comorbidities, self-reported health perception, age, gender, indigenous status, country of birth, language (other than English), marital status, home ownership, highest level of education, occupation and employment status, length and type of intervention; and baseline psychological distress or wellbeing, functional and structural social support, and physical function. Health (measured using the SF-36) and the preference based measure of health will be examined as predictors for count outcomes.

Adjustment will made for wave missing data (where a subject misses an entire visit) using inverse probability weighting [38]. This is a two-stage procedure. In the first stage a logistic regression model is used to predict which subjects are more likely to be missing at each follow-up time. This model will include time, demographic factors and comorbidity factors collected at baseline. The probability of each subject being missing at each time is then calculated and inverted to create a weight that can be used in the regression models described above. As an example, if men at 12-months have a 0.5 probability of responding, then the weight for men is 2 so that the results for those men who responded at 12-months are doubled. This will enable a sensitivity analysis with comparison to the complete case analysis to be conducted.

The health of the study sample (using SF-36 scores) will be compared to Australian normative data [39] matched on select sociodemographic variables such as age, gender and select chronic diseases using unpaired t-tests with 95% confidence intervals.

### Ethics

Each subject will provide written consent at baseline. Ethics approval has been granted by the Central Queensland Human Research Ethics Committee [HREC 11/QCQ/14].

## Discussion

This study is a broad evaluation of the client health outcomes and healthcare costs of people attending a unique regional service that is attempting to meet the health needs of regional and rural Australians with chronic disease. The study will provide preliminary evidence on the effectiveness of interdisciplinary intervention for this clinical population; an area that has received little investigation to date [8]. In addition, it will provide evidence regarding the influence of a range of variables including multimorbidity on the health and health costs of people with chronic disease in a rural and regional area. Comparison of the health state of people attending the service to the general population will determine the severity of health issues in the study population. The combined evidence will greatly assist in informing future service delivery and research.

The need for rigorous non-randomised studies in community settings has been highlighted by Bettger and Stineman [40] as these studies can identify target populations and appropriate outcomes for measurement that are important foundations for randomised controlled trials (or can even inform if randomised trials are required at all). Of relevance to the many participants with multimorbidity who are likely to be recruited in this study, randomised controlled trials to evaluate long-term outcomes have been discussed as inappropriate for people with conditions likely to worsen over time regardless of the intervention received [40]. Thus this longitudinal study will provide important information on the progressive impact of chronic disease in people in regional and rural areas who are involved in this unique intervention program. Specifically, the study will contribute knowledge about hospital use, health and healthcare costs associated with chronic disease for people in regional and rural areas over time. The factors that influence these changes will also be investigated.

Economic and health-related outcomes from this investigation will inform other health services throughout Australia (and elsewhere) who are considering adopting comparable models of service delivery. Outcomes from this study will also inform future research investigating health service needs and clinical needs for comparable populations.

## Abbreviations

BMI: Body mass index; SF-36: 36-item short-form health survey; K6: Kessler6; TUG: Timed up and go test.

## Competing interests

The authors declare they have no competing interests.

## Authors’ contributions

ZT conceived of the study, participated in its design, set up the study processes, will oversee data collection, and drafted the protocol. SM participated in the design of the study, statistical support and assisted in drafting the protocol. KF participated in the design and coordination of the study and assisted in drafting the protocol. PC and SK participated in the design of the study and assisted in drafting the manuscript. AGB contributed to statistical design and manuscript editing. All authors read and approved the final manuscript.

## Pre-publication history

The pre-publication history for this paper can be accessed here:

http://www.biomedcentral.com/1472-6963/13/410/prepub
